# Impact of Tissue Storage on Bioanalytical Data and Live Tissue Science

**DOI:** 10.1007/s11912-026-01864-6

**Published:** 2026-11-05

**Authors:** Erick Beltran, Ben Youngs, Luke Allen, Elliot Zinn, Bryson Leary, Emilee Goold, Andralyn McKell, Diana Nortey-Annan, Kaitlin Bailey, Lindsay Perrett, Myler Yee, DeLaney Anderson, Bridger Kearns, Hyrum Lakko, Wesley Walpole, Isaac Steveson, Alberto Trujano, Parker Romney, Ken Dixon, Jared Barrott

**Affiliations:** 1 Department of Cell Biology & Physiology, Brigham Young University, Provo, UT 84602 USA; 2 Simmons Center for Cancer Research, Brigham Young University, Provo, UT 84602 USA; 3 Specicare, 690 Medical Park Ln, Gainesville, GA 30501 USA; 4 School of Medicine, Brigham Young University, Provo, UT 84602 USA

**Keywords:** Biospecimen management, Precision medicine, Oncology, Cryopreservation, FFPE

## Abstract

**Purpose of Review:**

Precision oncology increasingly depends on accurate molecular and functional characterization of tumor tissue across genomic, transcriptomic, proteomic, epigenetic, and live-cell applications. This review evaluates how commonly used tissue preservation methods, including formalin-fixed paraffin-embedded (FFPE), fresh, flash-frozen, and cryopreserved tissue, affect downstream analytical fidelity and translational utility.

**Recent Findings:**

Recent comparative studies demonstrate that FFPE remains effective for histopathology and a few established molecular assays but introduces nucleic acid fragmentation, crosslinking, deamination artifacts, protein modification, and loss of cellular viability. These effects can reduce sequencing efficiency, alter quantitative molecular measurements, and limit applications requiring intact proteins or viable cells. In contrast, flash-frozen and cryopreserved tissues generally provide greater molecular fidelity, while controlled-rate cryopreservation additionally preserves viable cellular populations for single-cell analysis, organoid development, functional precision medicine, tumor-infiltrating lymphocyte expansion, and CAR-T manufacturing. Emerging evidence also supports the use of cryopreserved living tumor biobanks for longitudinal and functional testing.

**Summary:**

No single preservation method is optimal for every application. FFPE remains essential for routine pathology, whereas flash freezing is well suited for high-fidelity molecular profiling and controlled-rate cryopreservation offers the broadest compatibility with both molecular and live-cell applications. Prospective biospecimen strategies that align tissue preservation with anticipated diagnostic, molecular, functional, and therapeutic uses may improve the reliability and clinical utility of precision oncology.

## Introduction

Advances in precision oncology have increasingly relied on molecular characterization of tumor tissue to guide diagnosis, prognosis, therapeutic selection, and disease monitoring [1–3]. High-throughput sequencing, transcriptomic profiling, proteomics, and functional cellular assays now allow tumors to be evaluated across multiple biological layers, enabling a more comprehensive understanding of tumor heterogeneity and therapeutic vulnerability [4–7]. However, the accuracy of these downstream analyses depends heavily on the methods used to collect, preserve, and process biospecimens prior to molecular interrogation [8, 9].

Formalin-fixed paraffin-embedded (FFPE) tissue remains the standard preservation method in clinical pathology because it provides excellent histomorphologic preservation, long-term room-temperature stability, and compatibility with routine diagnostic workflows [10]. Despite these advantages, formalin fixation introduces chemical crosslinking, nucleic acid fragmentation, deamination artifacts, and protein modification that may compromise molecular fidelity. Although many analytical workflows have been optimized to accommodate FFPE-derived material, these adjustments frequently require shortened amplicons, increased amplification, computational artifact correction, or selective transcript targeting, each of which may introduce additional technical bias [11, 12].

Importantly, many FFPE optimization strategies are benchmarked against matched fresh or frozen tissue, which continues to serve as the reference standard for molecular fidelity [13, 14]. The consequences of preservation-induced molecular alteration vary across biological systems. DNA sequencing applications often demonstrate moderate discordance between FFPE and matched fresh or frozen tissue, whereas RNA sequencing, methylation profiling, and proteomic analyses exhibit progressively greater susceptibility to fixation-related degradation and distortion. These limitations become even more significant in live-cell applications, including organoid development, ex vivo drug screening, tumor-infiltrating lymphocyte (TIL) expansion, and CAR-T manufacturing, where preservation of cellular viability and function is essential.

In contrast, fresh tissue, flash freezing, and cryopreservation generally preserve molecular and cellular integrity more effectively by minimizing chemical modification and limiting enzymatic degradation [14, 15]. However, these approaches introduce logistical challenges related to storage, transport, infrastructure, and workflow integration. As precision oncology increasingly incorporates multiomic and functional assays into clinical decision-making, balancing practical feasibility with biological fidelity has become a critical consideration in biospecimen management.

Importantly, preservation-associated effects are highly context-dependent and may vary substantially across tissue types, tumor cellularity, ischemia intervals, fixation duration, storage conditions, extraction methodologies, sequencing platforms, and library preparation workflows [16, 17]. Differences in stromal composition, necrosis, nucleic acid abundance, and pre-analytical handling can significantly influence downstream molecular performance independent of preservation method alone [18]. Furthermore, advances in extraction chemistry, computational artifact correction, and optimized library preparation strategies continue to improve the analytical utility of FFPE-derived material in selected applications [19, 20]. Consequently, the magnitude of preservation-associated distortion observed across studies should be interpreted within the broader context of assay design, tissue handling protocols, and intended downstream clinical or research application.

**Fig. 1:**
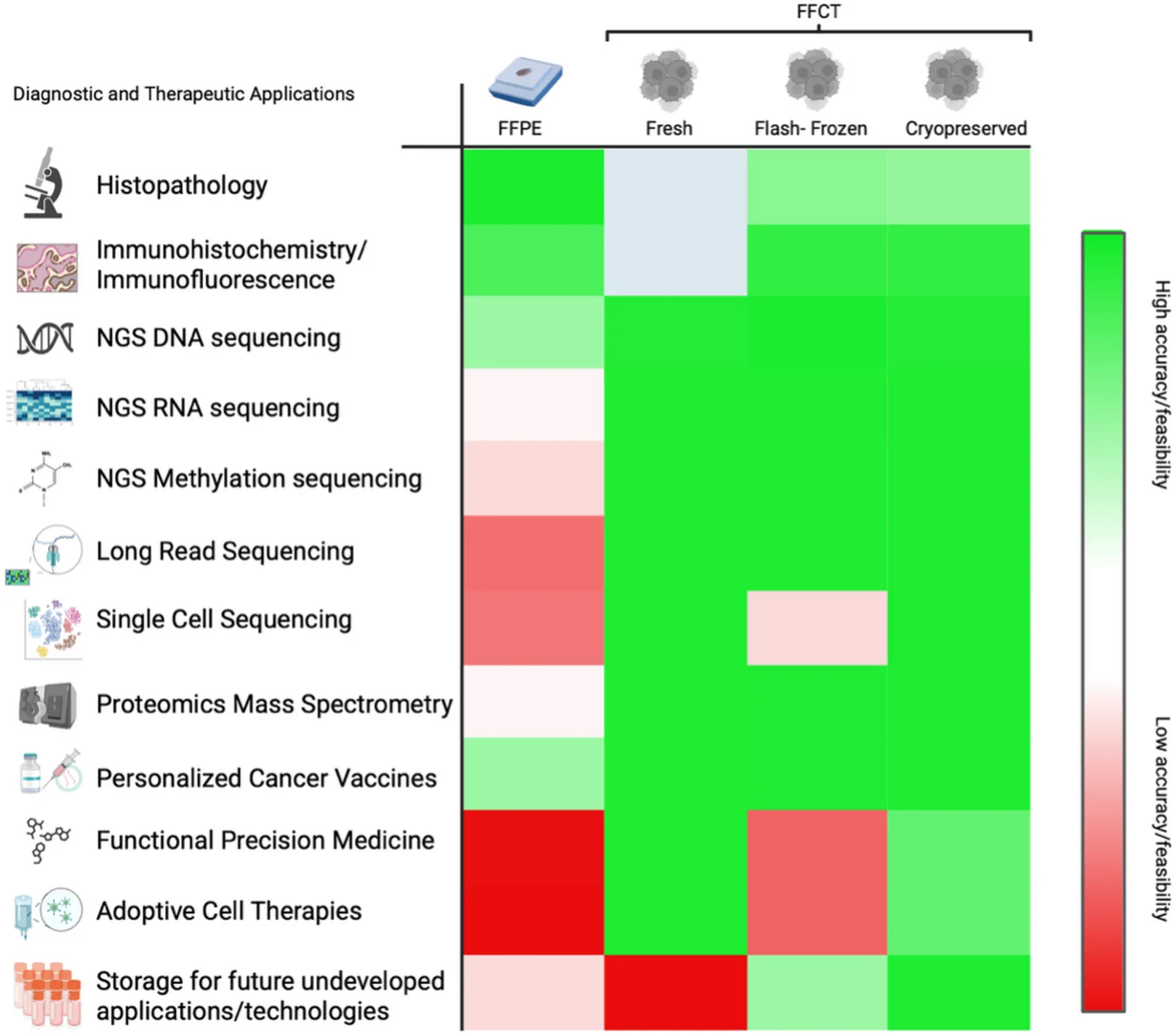
Accuracy and feasibility chart for different diagnostic and therapeutic applications across the tissue formats: FFPE, flash frozen, fresh, and cryopreserved. Red indicates a combination of low accuracy and/or feasibility, whereas green indicates high accuracy and/or feasibility. Colors were determined by comparative research studies and stand-alone feasibility studies working with the various tissue formats. FFPE remains highly capable for morphology and some mature molecular assays; flash freezing maximizes molecular fidelity; and controlled-rate cryopreservation provides the broadest compatibility when viable biological function must also be retained

This review evaluates the impact of tissue preservation methods across DNA sequencing, methylation profiling, transcriptomics, proteomics, and live tissue applications (Fig. 1). Particular emphasis is placed on how preservation-related artifacts influence downstream analytical accuracy, functional interpretation, and translational utility in modern precision medicine.

Literature was identified through PubMed searched using combinations of terms including FFPE, fresh frozen, cryopreservation, DNA sequencing, RNA sequencing, proteomics, DNA methylation, organoids, TILs, and CAR-T. Priority was given to original and direct comparative studies evaluating matched preservation methods in clinically relevant human biospecimens published within the past six years. Seminal older publications were included where necessary to provide historical context or describe foundational methodologies. Reference lists of relevant articles were also screened to identify additional studies not captured in the primary search.

## Main Text

### Histology and Immunohistochemistry

Histologic evaluation and immunohistochemistry (IHC) remain foundational tools in cancer diagnosis and biomarker assessment. FFPE tissue is widely favored in clinical pathology because formalin fixation preserves tissue architecture exceptionally well and enables long-term storage at room temperature. However, preservation of morphology does not necessarily equate to preservation of molecular fidelity. Numerous studies demonstrate that formalin fixation can reduce antigen detectability through protein crosslinking and epitope masking, thereby affecting the sensitivity and reproducibility of immunohistochemical analyses [13, 21].

Compared with matched FFPE tissue, fresh-frozen samples consistently demonstrate stronger and more reliable staining for several clinically relevant protein markers. Reduced antigen detectability in FFPE tissue has been reported for markers including CD31, CD68, HER2, p53, and PD-L1, even following antigen retrieval procedures [13, 22]. Importantly, these reductions do not necessarily reflect absence of the underlying protein, but rather decreased antibody accessibility secondary to fixation-induced conformational changes.

Several studies additionally demonstrate that antigen degradation in FFPE tissue may worsen over time, with prolonged storage contributing to declining staining sensitivity and increased false-negative rates for clinically actionable biomarkers [23]. Delayed fixation prior to formalin exposure has likewise been associated with reduced detection of hormone receptors and HER2, in some cases altering tumor classification and potentially influencing treatment eligibility [21].

For example, studies evaluating PD-L1 antibodies demonstrated reductions in sensitivity from 100% in frozen tissue to 67% and 33% following FFPE processing depending on antibody clone selection [22]. Although antigen retrieval techniques can partially improve signal recovery, restoration is incomplete and highly dependent on the target protein and retrieval protocol. Consequently, FFPE-derived IHC results may exhibit variable sensitivity across biomarkers, introducing additional analytical variability into diagnostic interpretation.

In contrast, fresh-frozen and cryopreserved tissues generally maintain protein conformations closer to their native biological state, improving epitope accessibility and molecular fidelity. However, these methods are associated with greater logistical complexity and often provide inferior morphologic preservation relative to FFPE [13, 21–23]. Accordingly, tissue preservation in histopathology represents a tradeoff between structural stability and molecular accuracy, with the optimal approach depending on the intended downstream application.

### Next Generation DNA Sequencing

Next generation sequencing (NGS) has become central to precision oncology by enabling rapid identification of somatic mutations, copy number alterations, and clinically actionable genomic variants [24–26]. Because sequencing accuracy depends heavily on nucleic acid quality, tissue preservation method represents a major determinant of downstream analytical performance [15, 26]. Fresh-frozen and cryopreserved tissues generally preserve DNA integrity more effectively than FFPE tissue, whereas formalin fixation introduces fragmentation, crosslinking, and chemical modification that can compromise sequencing fidelity [11, 27, 28].

Across diverse tumor types and sequencing platforms, FFPE-derived DNA consistently demonstrates reduced fragment length, lower amplification efficiency, decreased library complexity, and increased sequencing artifacts relative to matched flash frozen (FF) or cryopreserved tissue [11, 15, 27, 28]. These effects are particularly problematic in applications requiring high molecular fidelity, including whole genome sequencing (WGS), low-frequency variant detection, and long-read sequencing approaches [29]. Although optimized extraction protocols and bioinformatic correction methods can partially mitigate these limitations, preservation-associated artifacts continue to contribute to discordant variant calling and reduced reproducibility across platforms.

To compare preservation methods, this section synthesizes evidence across the major determinants of DNA sequencing performance, including DNA yield and fragment integrity, sequencing artifacts, variant concordance, and clinical workflow implications. These parameters collectively define the analytical quality of sequencing data and ultimately influence the clinical utility of genomic profiling.

#### DNA Yield and Fragment Integrity

The quality of DNA obtained from preserved tumor tissue is a major determinant of NGS performance. Preservation-associated fragmentation, reduced extraction yield, and impaired amplification efficiency can significantly affect library preparation, sequencing accuracy, and downstream variant detection. Across multiple studies, FF and cryopreserved tissues consistently outperform FFPE tissue with respect to DNA integrity, fragment length, and sequencing feasibility [11, 15, 27, 28].

One of the primary limitations of FFPE-derived DNA is extensive fragmentation caused by formalin fixation and long-term storage. Compared with FF and cryopreserved tissue, FFPE samples consistently yield shorter DNA fragments, reducing the number of viable templates available for amplification and library construction. In direct comparisons, FFPE-derived DNA fragment insert size averaged 391–444 bp, whereas matched FF and cryopreserved samples yielded substantially longer fragment insert sizes ranging from approximately 477–645 bp [11, 15]. In some cryopreserved samples, DNA fragments greater than 50,000 bp were preserved, supporting compatibility with long-read sequencing approaches and WGS applications [27]. Shorter fragment lengths in FFPE additionally increase the likelihood of chimeric reads and incorrect genomic mapping, further compromising sequencing fidelity.

These structural differences have important downstream effects on PCR amplification efficiency and library preparation success. In studies evaluating targeted amplification of EGFR and KRAS exons, FF samples achieved nearly universal PCR success, whereas matched FFPE samples demonstrated amplification success rates closer to 50% [12]. The type of formalin fixative also influenced performance, as neutral buffered formalin outperformed simple formalin, likely because of improved pH stability during fixation. Reducing amplicon size from 295 bp to 235 bp substantially improved amplification success in FFPE-derived DNA, supporting the conclusion that preservation-associated fragmentation is a major contributor to sequencing failure in FFPE tissue [12].

Because PCR amplification occurs exponentially during library preparation, even modest reductions in amplification efficiency can substantially decrease sequencing depth and coverage uniformity. These effects are particularly problematic in low-input clinical samples such as fine-needle biopsies, where limited starting material already constrains sequencing performance. In clinical studies, freshly frozen fine-needle biopsies achieved a 99% success rate for obtaining sufficient DNA input for WGS despite tumor purities as low as 20% [30, 31].

Quantitative comparisons similarly demonstrate substantial differences in sequencing yield between preservation methods. Bhagwate et al. reported that FF tissue yielded DNA fragments approximately five times longer and generated approximately 80% greater DNA yield than matched FFPE samples [32]. Likewise, Oba et al. demonstrated that even optimized FFPE extraction workflows produced markedly lower library yields and sequencing coverage than FF-derived DNA. Using equivalent 30 ng DNA inputs, FF tissue generated approximately tenfold greater library yield and substantially higher mean coverage than FFPE tissue [33]. Cryopreserved tissue also demonstrated significantly greater DNA yield and fragment preservation relative to FFPE, further supporting its utility for applications requiring high molecular integrity [27].

Although specialized extraction methods and shorter amplicon strategies can partially mitigate these challenges, such adjustments increase analytical complexity and may introduce additional bias. In contrast, FF and cryopreserved tissues preserve sufficient DNA integrity to support robust library preparation, deeper sequencing coverage, and broader compatibility with whole genome and long-read sequencing applications with less starting material, thus favoring small biopsies.

#### Sequencing Artifacts and Variant Discordance

In addition to reducing DNA yield and fragment integrity, FFPE preservation introduces chemical alterations that can significantly affect sequencing accuracy and downstream variant interpretation. Formalin fixation is associated with cytosine deamination, oxidative damage, and nucleotide crosslinking, all of which contribute to sequencing artifacts that are substantially less common in FF and cryopreserved tissues [34, 35]. These preservation-induced alterations can generate false variant calls, distort variant allele frequencies, and reduce concordance between matched samples.

One of the most well-characterized FFPE-associated artifacts is cytosine deamination, which contributes to characteristic C > T/G > A substitution patterns during sequencing analysis [36]. Studies comparing matched FFPE and FF samples consistently demonstrate increased artifact burden in FFPE-derived DNA, even when overall mapping statistics appear similar between preservation methods. In a direct comparison using the breast cancer cell line HCC1395, mapped reads and nonredundant read frequencies were comparable between FFPE and fresh samples; however, precision and recall during variant calling were substantially reduced in FFPE-derived DNA because of fixation-associated nucleotide artifacts [26]. Importantly, some FFPE-associated artifacts exceeded variant allele frequency (VAF) thresholds commonly used for bioinformatic filtering, complicating efforts to distinguish true low-frequency somatic mutations from preservation-induced sequencing noise [26].

Early work demonstrated artifact frequencies approaching one sequence alteration per 500 base pairs in FFPE-derived DNA [37], providing some of the first evidence that formalin fixation can introduce sequence changes detectable by molecular assays. Subsequent studies demonstrated clinically meaningful discordance between FFPE and matched FF tumor samples, including discrepancies in KRAS and TP53 variant detection [38, 39]. In metastatic colorectal cancer, direct comparison of matched FFPE and FF samples revealed greater than 27% discordance in KRAS mutation calls, including both false wildtype classifications and discrepancies in specific mutation identity [38]. Similarly, studies evaluating colorectal tumors with targeted gene panels identified numerous variants unique to FFPE samples, with reported TP53 concordance rates falling below 75% [39]; however, the recalculated concordance rate was 55% once baseline negatives were removed from the equation. In melanoma whole-exome sequencing studies, FFPE samples exhibited extensive library preparation failures, loss of thousands of detectable variants, and concordance rates as low as 43%, largely attributable to low DNA quality and fixation-associated artifact burden [29].

Large-scale sequencing studies further support the conclusion that FFPE processing substantially increases genomic distortion. In a multicenter analysis of 2,520 FF samples collected across 44 hospitals, only 0.11% of samples demonstrated severe global imbalance value scores indicative of DNA damage. In contrast, retrospective analyses of FFPE-rich databases such as The Cancer Genome Atlas and the 1,000 Genomes Project identified severe imbalance scores in 41% and 73% of samples, respectively [40]. These findings highlight the broad impact of formalin fixation on genomic integrity across large sequencing cohorts.

FFPE-associated artifacts also influence quantitative genomic metrics such as tumor mutational burden (TMB) and mutant-allele heterogeneity scores. In paired gastroesophageal tumors analyzed by whole-exome sequencing, FFPE specimens demonstrated significantly higher mutant-allele heterogeneity scores than matched fresh-frozen tissue, suggesting that fixation-associated artifacts may influence quantitative measures of genomic complexity [41, 42]. These findings are particularly relevant in precision oncology, where TMB is increasingly used to guide immunotherapy eligibility and therapeutic stratification.

Although advances in extraction protocols, shorter amplicon strategies, DNA repair methods, and computational filtering have substantially improved sequencing performance, fixation-induced nucleotide alterations continue to reduce variant concordance and complicate interpretation of clinically actionable mutations. In contrast, fresh-frozen and cryopreserved tissues consistently demonstrate greater genomic fidelity and lower artifact burden, supporting more accurate detection of somatic variants and increasing confidence in downstream clinical interpretation. These differences become increasingly important as genomic profiling assumes a greater role in biomarker-guided therapy selection, highlighting the close relationship between biospecimen quality and the clinical utility of molecular diagnostics.

#### Clinical Consequences of Preservation

The technical limitations introduced by tissue preservation methods have direct consequences for clinical sequencing workflows and patient care. Reduced DNA integrity, failed library preparation, low sequencing yield, and preservation-associated artifacts can increase assay failure rates, prolong turnaround times, and delay initiation of biomarker-guided therapies, which may ultimately contribute to suboptimal therapeutic decision-making.

FFPE-derived DNA frequently requires additional optimization during library preparation because fragmentation and reduced amplification efficiency compromise sequencing performance. Failed amplification reactions, insufficient library complexity, uneven coverage, and low read depth often necessitate repeat extraction, repeat sequencing, or redesign of assay parameters using shorter amplicons [12, 33]. These additional troubleshooting steps increase workflow complexity and reduce reproducibility, particularly in low-input clinical specimens such as fine-needle biopsies.

The impact of preservation-associated sequencing failure becomes especially important in clinical oncology settings where treatment decisions are increasingly dependent on timely molecular profiling. Even in highly optimized sequencing programs, turnaround times for comprehensive genomic testing may approach two weeks. A recent clinical study reported an average turnaround time of 13.9 days for lung cancer molecular profiling despite substantial workflow optimization efforts [43]. In less optimized clinical environments, preservation-associated library failures and repeat testing may further extend these timelines.

These delays have meaningful implications for patient management. Several studies demonstrate that a substantial proportion of patients initiate systemic therapy before comprehensive molecular profiling is available, reducing the likelihood that first-line treatment decisions can be informed by the complete genomic landscape of the tumor [44, 45]. In advanced non-small cell lung cancer, approximately 24% of patients initiated therapy prior to receipt of genomic profiling results [44]. Broader analyses of oncology workflows suggest that up to 50% of patients may begin first-line treatment without complete biomarker testing, with some tumor types exhibiting rates as high as 83% [45]. Delayed molecular results may therefore contribute to suboptimal treatment selection, missed eligibility for targeted therapies, and reduced implementation of precision oncology strategies.

Importantly, many of these workflow limitations are closely tied to the reduced molecular fidelity of FFPE-derived DNA. Lower sequencing yield, increased artifact burden, and higher assay failure rates collectively increase the likelihood of repeat processing and delayed reporting. In contrast, FF and cryopreserved tissues generally provide higher-quality DNA with greater amplification success, more reliable library preparation, and improved sequencing consistency, thereby supporting more efficient downstream clinical workflows [26, 32, 42].

These findings suggest that preservation strategy influences not only sequencing accuracy, but also the practical implementation of precision medicine in clinical care. As molecular profiling becomes increasingly central to therapeutic decision-making, reducing preservation-associated sequencing failures may improve both analytical reliability and the timeliness of patient-specific treatment selection.

### Next Generation Sequencing DNA Methylation

DNA methylation profiling has become increasingly important for tumor classification, prognostic assessment, biomarker discovery, and emerging precision oncology applications, making preservation-related analytical bias an important consideration for both research and clinical testing [46]. Accurate methylation analysis depends on preservation of native cytosine methylation patterns throughout tissue processing and nucleic acid extraction. However, preservation-induced DNA damage and chemical modification can alter methylation measurements and reduce confidence in epigenomic interpretation [47].

Several studies comparing FFPE and FF tissues demonstrate that formalin fixation can reduce the accuracy of methylation measurements through DNA fragmentation, crosslinking, reduced template quality, and amplification bias introduced during downstream analytical workflows [46, 47]. Although global methylation patterns often remain broadly correlated between preservation methods, FFPE samples consistently exhibit reduced DNA quality and increased variability in methylation measurements at specific CpG loci [46–48].

Comparative analyses of colorectal carcinoma specimens have demonstrated that FFPE-derived DNA can exhibit increased variability in methylation measurements relative to matched FF tissue, with the magnitude of these differences depending on both the target locus and the DNA extraction methodology [48]. Similar findings have been observed in ovarian and pancreatic cancer studies, where FFPE samples showed reduced technical quality scores, higher probe failure rates, and increased deviation from matched fresh tissue methylation profiles. In one ovarian cancer methylation study, 87.4% of failed probes originated from FFPE-derived tissue, whereas only 2.72% of failed probes were associated with FF samples, suggesting substantially greater preservation-associated technical variability in FFPE specimens [49].

Importantly, preservation-associated methylation analytical variability may disproportionately affect low-quality or region-specific analyses, particularly in studies focused on individual biomarker loci rather than broad global methylation trends. Consequently, validation between FFPE and fresh or frozen tissue may be necessary when methylation status is used for diagnostic, prognostic, or therapeutic decision-making.

In summary, preservation-associated effects extend beyond DNA sequence alone and influence epigenomic characterization as well. Although FFPE remains suitable for some methylation applications, FF tissue generally provides greater confidence in quantitative methylation analyses and may be preferable when individual methylation biomarkers are used for clinical decision-making.

### Next Generation Sequencing RNA Transcriptomics

The effects of tissue preservation become substantially more pronounced when moving from DNA sequencing to transcriptomic analysis because RNA is inherently less stable than DNA and is highly susceptible to enzymatic degradation and chemical modification. As a result, tissue preservation is a critical determinant of RNA sequencing quality, transcript quantification, and downstream biological interpretation. The growing clinical importance of RNA sequencing extends beyond differential gene expression analysis to include molecular subtyping, immune profiling, fusion transcript detection, pathway analysis, and biomarker discovery. Consequently, preservation-induced degradation can influence not only analytical performance but also the biological interpretation of tumor state and therapeutic vulnerability.

To evaluate how tissue preservation influences RNA sequencing performance, this section synthesizes evidence across the major determinants of transcriptomic fidelity, including RNA integrity, sequencing performance, preservation of viable cellular populations, and downstream clinical utility. These characteristics collectively define the analytical quality of transcriptomic data and determine the suitability of preserved tissue for both bulk and single-cell RNA sequencing applications.

#### RNA Integrity and Sequencing Performance

RNA integrity is one of the strongest predictors of transcriptomic data quality. Fresh tissue provides the biological reference standard for transcriptomic analyses, while flash freezing most closely preserves the native transcriptome by rapidly stabilizing RNA and minimizing post-excision degradation. FF tissue consistently demonstrates high RNA integrity numbers (RIN ~ 8.0–8.1), high DV200 values (~ 96–97%), preservation of long transcript fragments, and reliable exon-aligned sequencing with minimal mapping bias [14, 50, 51]. In one comparative study, FFPE-derived samples demonstrated approximately 13% unmapped sequencing reads compared with only 4% in matched FF tissue, highlighting the substantial impact of preservation on sequencing quality [51]. Similarly, a pan-cancer comparison demonstrated approximately 15% fewer usable sequencing reads in FFPE specimens than matched FF tissue [52].

FFPE preservation introduces RNA fragmentation, formaldehyde-induced crosslinking, and chemical modification that reduce transcript length and compromise sequencing quality. FFPE-derived RNA consistently demonstrates markedly lower RIN values, reduced transcript detection sensitivity, increased proportions of unmapped reads, and greater representation of intronic or degraded RNA fragments [14, 50]. Although global gene expression profiles frequently remain moderately to highly correlated between matched FFPE and fresh tissue, fixation-associated degradation disproportionately affects low-abundance transcripts, fusion transcript detection, transcript isoform analysis, and quantitative expression measurements, thereby reducing the biological resolution achievable from FFPE-derived RNA.

The influence of preservation is illustrated in glioblastoma, where matched FFPE samples demonstrated substantially reduced RNA quality (2–3 times lower) and abundance compared to FF tissue, regardless of storage time. Although successful RNA extraction was markedly lower in FFPE specimens, transcriptomic classification remained feasible after restricting analyses to a curated gene panel, demonstrating that analytical optimization can partially compensate for reduced RNA quality but does not fully restore native transcriptomic fidelity [53].

#### Preservation of Transcriptomic Fidelity and Single-Cell Applications

Beyond preserving RNA integrity, modern transcriptomic analyses increasingly require maintenance of viable cellular populations for single-cell sequencing. Unlike FF tissue, which disrupts cellular membranes during freezing, controlled-rate cryopreservation preserves both RNA integrity and cellular viability, enabling downstream single-cell RNA sequencing and functional analyses.

Multiple studies demonstrate that cryopreserved tissues maintain strong transcriptomic concordance with fresh tissue while preserving cellular heterogeneity and enabling accurate identification of immune, stromal, and malignant cell populations [54, 55]. Strong linear relationships between sequencing depth and transcript detection have likewise been observed for cryopreserved and fresh samples, indicating minimal loss of transcriptomic sensitivity following cryopreservation [54]. Although modest activation of stress-response pathways may occur during freeze-thaw cycling, these transcriptional changes generally represent a small proportion of the overall transcriptome and have minimal effect on global biological interpretation.

Importantly, recent studies have demonstrated that slow-frozen cryopreserved tumor tissue remains suitable for organoid development, ex vivo culture, and single-cell RNA sequencing while preserving both cancer and stromal cell populations. Although minor differences in cellular proportions have been reported, the principal transcriptomic differences were largely confined to heat-shock response pathways, indicating that controlled cryopreservation closely approximates fresh tissue for many downstream functional applications [55].

#### Clinical Implications for Transcriptomic Analysis

The increasing clinical adoption of RNA sequencing has expanded the importance of tissue preservation beyond research applications alone. Molecular subtyping, immune profiling, gene fusion detection, and expression-based biomarkers increasingly influence therapeutic decision-making, making accurate transcript quantification essential for precision oncology.

Although modern targeted capture methods and specialized library preparation protocols have substantially improved the feasibility of RNA sequencing from FFPE-derived tissue, these approaches primarily compensate for degraded RNA rather than restoring native transcript integrity [50]. Consequently, FFPE-derived RNA frequently requires increased sequencing depth, transcript filtering, and analytical correction to achieve performance comparable with FF specimens. These additional processing steps increase analytical complexity and may reduce sensitivity for quantitative transcriptomic analyses.

Collectively, the literature demonstrates that preservation-associated effects become substantially more pronounced when moving from genomic to transcriptomic analyses. While FFPE remains an important resource for retrospective studies and archival specimens, FF tissue continues to provide the highest transcriptomic fidelity, and controlled-rate cryopreservation offers the closest approximation to fresh tissue while uniquely preserving viable cellular populations for single-cell and functional applications. As precision oncology increasingly incorporates RNA-based diagnostics and live-cell technologies, preservation strategies that maintain both transcript integrity and cellular viability will become increasingly important for maximizing the clinical utility of transcriptomic profiling.

### Proteomics

While genomic and transcriptomic analyses characterize the molecular blueprint of a tumor, proteomic analyses provide insight into the functional state of the cell by measuring protein abundance, post-translational modifications, and signaling pathway activity. Because proteins are structurally dynamic and chemically diverse, preservation-induced alterations can have a disproportionately large impact on downstream analytical performance and biological interpretation.

To evaluate the influence of tissue preservation on proteomic analyses, this section synthesizes evidence across the principal determinants of proteomic fidelity, including protein integrity and detection depth, preservation-induced analytical artifacts, and maintenance of functional signaling pathways. Together, these characteristics determine the suitability of preserved tissue for quantitative proteomics and phosphoproteomic analyses.

#### Protein Integrity and Detection Depth

**Fig. 2:**
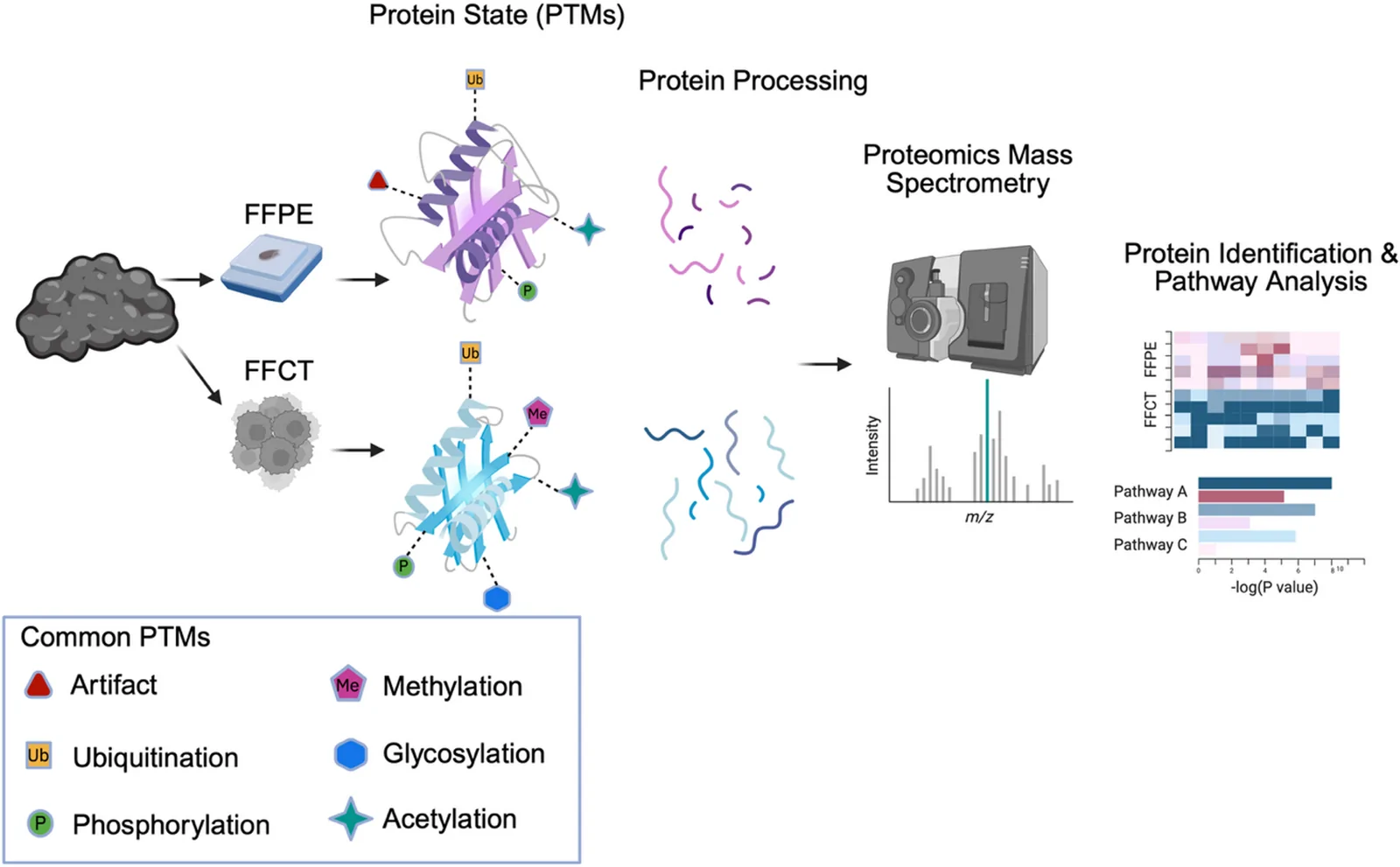
Effects of tissue preservation on proteomic analysis. FFPE processing can introduce crosslinking, artifactual post-translational modifications, and reduced peptide recovery, potentially altering protein-expression and pathway analyses. Fresh, flash-frozen, and cryopreserved tissues generally retain greater proteomic fidelity, although performance varies by tissue type and preservation protocol. FFCT = fresh, flash-frozen, and cryopreserved tissue

Preservation of native protein structure is fundamental for accurate mass spectrometry-based proteomic analyses. FF tissue consistently provides the highest proteomic fidelity by minimizing protein degradation, maintaining native conformational states, and preserving post-translational modifications (PTMs) present at the time of tissue collection (Fig. 2). Across multiple studies, FF tissue demonstrates greater proteome depth, improved phosphoproteomic detection, and more reliable identification of PTMs than matched FFPE specimens [56, 57].

Quantitative comparisons further illustrate the effects of preservation on protein recovery. Using liquid chromatography-tandem mass spectrometry (LC-MS/MS), FF specimens yielded approximately 1,511 identifiable proteins per sample compared with only 947 proteins recovered from matched FFPE tissue [57]. The reduced detection depth observed in FFPE specimens limits characterization of low-abundance proteins and decreases sensitivity for pathway-level analyses, particularly when limited tissue is available.

#### Preservation-Induced Proteomic Artifacts

Unlike nucleic acid degradation, the principal effects of formalin fixation on proteins arise from extensive intermolecular crosslinking and chemical modification. Formaldehyde reacts with amino acid side chains and terminal amino groups to form covalent crosslinks that alter protein conformation, reduce solubility, and modify peptide masses detected during mass spectrometry. These preservation-induced alterations can obscure native PTMs and generate artificial molecular signatures that complicate biological interpretation [58].

Evidence of these analytical limitations has been demonstrated in pancreatic adenocarcinoma xenograft models, where FFPE specimens exhibited substantially greater numbers of apparent histone modification sites and additional PTMs than matched FF tissue. These findings suggest that fixation-induced chemical alterations may mimic endogenous PTMs and increase the difficulty of distinguishing biological signaling events from preservation artifacts [58].

Additional evidence for reduced proteomic fidelity is provided by Terminal Amine Isotopic Labeling of Substrates (TAILS), which evaluates protein N-termini following preservation. FF tissue yielded approximately 3,000–3,800 detectable N-termini compared with only 2,000–2,300 in matched FFPE specimens [59]. Although computational filtering and improved extraction methods can reduce analytical noise and improve peptide recovery, these approaches cannot fully reverse fixation-induced protein crosslinking or restore native protein structure [60]. Consequently, FFPE-derived proteomic analyses generally demonstrate reduced detection depth and greater analytical variability than FF tissue.

#### Functional Proteomics and Clinical Implications

Beyond protein identification alone, modern proteomic analyses increasingly seek to characterize dynamic signaling networks through phosphoproteomics and kinase activity profiling. Because phosphorylation and other post-translational modifications reflect functional cellular states, preservation of these molecular signatures is essential for understanding tumor biology and identifying therapeutic targets.

Controlled-rate cryopreservation has emerged as a promising strategy for preserving dynamic proteomic states while providing greater logistical flexibility than fresh tissue. Several studies demonstrate that phosphoproteomic and kinase signaling profiles remain remarkably stable during controlled cryopreservation and clinically relevant cold ischemia intervals [61]. In colorectal tumor specimens subjected to cold ischemia times of up to 180 min before preservation, only 90 of approximately 2,715 detected phosphopeptides exhibited significant changes in signal intensity, indicating that the vast majority (~ 97%) of phosphoproteomic features remained stable throughout the collection interval [61]. Similarly, MALDI imaging mass spectrometry demonstrated minimal proteomic differences between FF tissue and specimens stored at 4 °C for short periods prior to preservation, although prolonged ischemia eventually introduced measurable alterations [62].

The preservation of functional protein signaling has important implications for precision oncology. Proteomic and phosphoproteomic biomarkers are increasingly being incorporated into studies of therapeutic response, pathway activation, and drug target identification. Preservation-induced alterations in protein conformation or signaling activity therefore have the potential to influence both biomarker quantification and biological interpretation. Although modern extraction workflows have substantially improved the analytical utility of FFPE-derived specimens, FF tissue remains the reference standard for quantitative proteomics, while controlled-rate cryopreservation provides a practical alternative that preserves many functional signaling characteristics together with viable tissue for downstream analyses.

Collectively, these findings demonstrate that preservation-associated effects become increasingly consequential at the proteomic level because proteins are structurally and functionally more sensitive than nucleic acids. As precision oncology expands beyond genomic characterization toward functional molecular profiling, preservation strategies that maintain native protein structure and signaling activity will become increasingly important for accurate biomarker assessment, therapeutic target identification, and clinically meaningful biological interpretation.

### Personalized Cancer Vaccine Development

The preceding sections demonstrate that tissue preservation influences molecular characterization across DNA sequencing, transcriptomics, and proteomics. Personalized cancer vaccine development represents an important transition from measuring these molecular features to using them to guide individualized therapeutic strategies. Neoantigen discovery depends on accurate identification of tumor-specific mutations and confirmation of their expression, making preservation of high-quality tumor DNA and RNA essential for reliable vaccine target selection. Preservation-related errors at this stage may propagate through the translational workflow, affecting the identification, prioritization, and validation of candidate neoantigens. Although the final objective is therapeutic—to induce or expand immune responses capable of recognizing and eliminating tumor cells—the process begins with accurate molecular characterization of the tumor. Personalized cancer vaccines therefore provide a useful example of how biospecimen preservation can influence multiple stages of a translational workflow, linking molecular fidelity during specimen analysis with downstream therapeutic development. This framework also provides a transition to subsequent applications in which preservation must increasingly maintain viable cellular systems, functional responses, and therapeutic potency.

#### Preservation for Neoantigen Discovery

Personalized neoantigen vaccine development begins with identification of tumor-specific alterations that are absent from the patient’s normal tissues. Tumor DNA is therefore analyzed alongside matched-normal DNA, commonly obtained from peripheral blood, to distinguish somatic mutations from inherited variants. Candidate neoantigens are then prioritized according to factors such as variant allele frequency, tumor clonality, predicted major histocompatibility complex (MHC) binding, and evidence of mutant transcript expression [63, 64]. Because several of these parameters depend on accurate DNA and RNA measurements, preservation-related degradation or sequencing artifacts may affect both the detection of candidate variants and their subsequent prioritization.

Specimen quality directly affects this process. DNA degradation, sequencing artifacts, low tumor cellularity, or inadequate matched-normal data can produce false-positive or false-negative variant calls. Although FFPE tissue is frequently used because of its clinical availability, formalin-associated fragmentation, crosslinking, and nucleotide damage may complicate the detection of low-frequency or subclonal mutations and increase the potential for preservation-related false-positive or false-negative variant calls. Nevertheless, FFPE specimens remain suitable for many sequencing workflows when appropriately processed and analyzed. The relevant distinction is therefore not between usable and unusable tissue, but between preservation strategies that provide different levels of molecular fidelity and downstream flexibility. Fresh-frozen or appropriately cryopreserved tissue generally provides higher-quality nucleic acids for integrated DNA- and RNA-based analyses and may therefore be preferable when comprehensive neoantigen discovery is anticipate.

Tumor RNA sequencing further refines candidate selection by confirming that a mutation is expressed and by identifying alternative transcripts, fusions, retained introns, or abnormal splice junctions that may generate additional antigens. Tumor purity and spatial heterogeneity also matter because stromal contamination may reduce detection sensitivity, while regionally restricted mutations may not provide broad tumor coverage. Representative sampling, pathological review, tumor enrichment, and integration of DNA and RNA evidence are therefore essential for accurate vaccine design [65, 66].

#### Vaccine Manufacturing and Product Preservation

Selected neoantigens can be incorporated into synthetic long-peptide, mRNA, DNA, viral-vector, or dendritic-cell vaccines, each with distinct manufacturing and preservation requirements [64]. At this stage, preservation shifts from maintaining the integrity of the diagnostic biospecimen to maintaining the stability, identity, and functional capability of the individualized therapeutic product. This distinction is important because the preservation requirements of the final vaccine are platform-specific and are not necessarily equivalent to those of the tumor specimen used for neoantigen discovery.

Peptide vaccines are relatively stable but require individualized synthesis, purification, identity testing, and release assessment. Personalized mRNA vaccines can encode multiple neoantigens within a single construct and may allow faster redesign, but RNA must be protected from degradation and is commonly maintained in controlled frozen formulations. Preservation of both RNA integrity and delivery-particle characteristics is essential for maintaining antigen expression and immunogenicity [67].

DNA vaccines are more chemically stable than RNA but depend on efficient cellular uptake and antigen expression. Viral vectors can provide strong expression but introduce additional production, biosafety, and immunity considerations.

Dendritic-cell vaccines have the most stringent preservation requirements because they contain viable autologous cells. The final product must retain viability, antigen-presentation capacity, maturation phenotype, and T-cell–priming activity. Controlled-rate cryopreservation can support batch manufacturing, release testing, transport, and repeated dosing, but post-thaw evaluation should include recovery and functional potency rather than viability alone [68, 69]. Thus, personalized cancer vaccine development illustrates two distinct preservation-dependent stages of translation: preservation of tumor-derived molecular information for accurate target identification and preservation of the therapeutic product for safe and effective clinical administration.

#### Clinical and Translational Implications

The major translational challenge is completing an individualized workflow within a clinically meaningful interval. This process includes tissue acquisition, sequencing, HLA typing, neoantigen prediction, vaccine design, manufacturing, quality testing, and treatment delivery. Poor tissue quality or insufficient RNA can delay the process and may be especially consequential in patients with rapidly progressive disease.

Neoantigen prediction also remains imperfect. Predicted MHC binding does not guarantee that a peptide will be naturally processed, presented, or recognized by an effective T-cell repertoire. Candidate selection increasingly incorporates RNA expression, clonality, antigen-processing predictions, and immunologic validation, but only a subset of predicted neoantigens typically induces measurable immunity [63].

Early clinical studies have shown that personalized peptide and RNA vaccines can generate neoantigen-specific CD4-positive and CD8-positive T-cell responses in melanoma, glioblastoma, pancreatic cancer, and other malignancies [70–72]. In pancreatic ductal adenocarcinoma, a personalized mRNA vaccine produced expanded neoantigen-specific T-cell responses in a subset of patients, demonstrating the feasibility of individualized vaccine production during postoperative therapy [71].

Combination with immune-checkpoint blockade may further enhance activity by generating tumor-specific T cells while reducing inhibitory signaling within the tumor microenvironment. Early studies support the feasibility of this approach, although larger controlled trials are needed to establish consistent clinical benefit [70, 73, 74].

In summary, personalized cancer vaccines connect preservation of molecular information with generation of functional antitumor immunity. Their success depends on accurate tumor and matched-normal sequencing, confirmation of mutant expression, efficient manufacturing, and preservation of the final vaccine product.

### Functional Precision Medicine and Live Tissue Science

Functional precision medicine uses viable patient-derived cells or tissues to measure treatment response directly and complement predictions based on molecular biomarkers. These approaches include short-term cultures, tissue slices, organoids, and patient-derived xenografts. Because preservation-associated injury can alter cell recovery, cellular composition, and apparent drug sensitivity, preservation quality is central to the reliability of these assays.

#### Preservation of Viable Tissue

Fresh tumor tissue remains the biological reference standard for functional testing because it most closely preserves the cellular composition and physiological state present at resection. Patient-derived organoids established from fresh specimens can retain histologic, genomic, transcriptomic, and pharmacologic features of the parental tumor [75–77]. However, fresh tissue requires rapid transport and immediate processing, limiting use across geographically distributed clinical sites.

Controlled-rate cryopreservation offers greater logistical flexibility by separating tissue acquisition from downstream processing. Slow-frozen tumor tissue has supported generation of two-dimensional cultures, three-dimensional organoids, ex vivo cultures, and single-cell RNA sequencing, with results broadly comparable to freshly processed material [78]. Cryopreserved living tumor specimens have also retained viable malignant cells, tissue morphology, and sufficient functional integrity for later drug-sensitivity testing [79]. Organoids derived from cryopreserved ovarian cancer tissue similarly preserved major histologic, genomic, and transcriptomic characteristics of the source tumors [80].

Cryopreservation does not preserve all cellular components without change. Freeze-thaw injury may reduce total cell recovery, alter the relative abundance of fragile cell populations, and induce transient stress responses. Functional validation should therefore assess not only viability, but also tumor-cell representation, stromal and immune composition, proliferative capacity, and model establishment efficiency.

#### Preservation of Therapeutic Response

The most clinically important criterion is whether preserved tissue reproduces the therapeutic response of fresh material. In comparative studies, organoids generated from slow-frozen, DMSO-preserved tissue demonstrated morphology, metabolic activity, and drug-response patterns that more closely approximated fresh-derived organoids than those generated from flash-frozen tissue [81]. Slow-frozen colorectal tumor tissue has likewise produced organoids with growth and viability characteristics comparable to fresh specimens [78].

Cryopreserved ovarian cancer specimens also generated organoids whose drug-response profiles were associated with clinical treatment outcomes, although culture conditions influenced predictive performance [80]. These findings indicate that preservation is only one source of variability; ischemia time, tumor cellularity, dissociation method, culture medium, assay duration, and drug exposure conditions can also influence apparent therapeutic response.

FFPE tissue is incompatible with direct functional testing because fixation eliminates cellular viability. Flash freezing may occasionally permit organoid recovery, but its performance appears less predictable than controlled-rate cryopreservation for applications requiring reproducible cell recovery and drug response [81].

#### Clinical Implementation

Functional precision medicine is intended to complement molecular profiling. Genomic analysis may identify potentially actionable alterations, whereas functional testing measures whether patient-derived tissue responds to therapy in the integrated biological context of the tumor. This combination may be particularly useful when molecular findings are ambiguous or multiple treatment options are available.

Prospective studies support the feasibility of organoid-guided testing within clinically relevant timelines. In advanced pancreatic cancer, organoid screening identified at least one potentially active treatment for most evaluable patients, although prospective interventional validation remains necessary before routine clinical adoption [82]. Associations between organoid drug sensitivity and clinical response have also been reported in breast, colorectal, bladder, and ovarian cancers [75, 76, 80, 83].

Cryopreservation may expand access by enabling centralized testing, multicenter studies, viable tissue biobanking, and later reassessment as new therapies become available. These advantages must be balanced against the need for controlled freezing, monitored storage, standardized thawing, and documentation of pre-analytical variables.

Collectively, the evidence indicates that preservation requirements change fundamentally when the goal shifts from measuring molecular composition to evaluating living tumor behavior. Fresh tissue provides the closest representation of the immediate biological state, while controlled-rate cryopreservation currently offers the most practical alternative for preserving viable tissue and clinically relevant therapeutic-response patterns. This transition from molecular fidelity to functional fidelity also establishes the framework for TIL isolation and engineered T-cell manufacturing.

### Tumor-Infiltrating Lymphocyte Therapy and Live Tissue Science

**Fig. 3:**
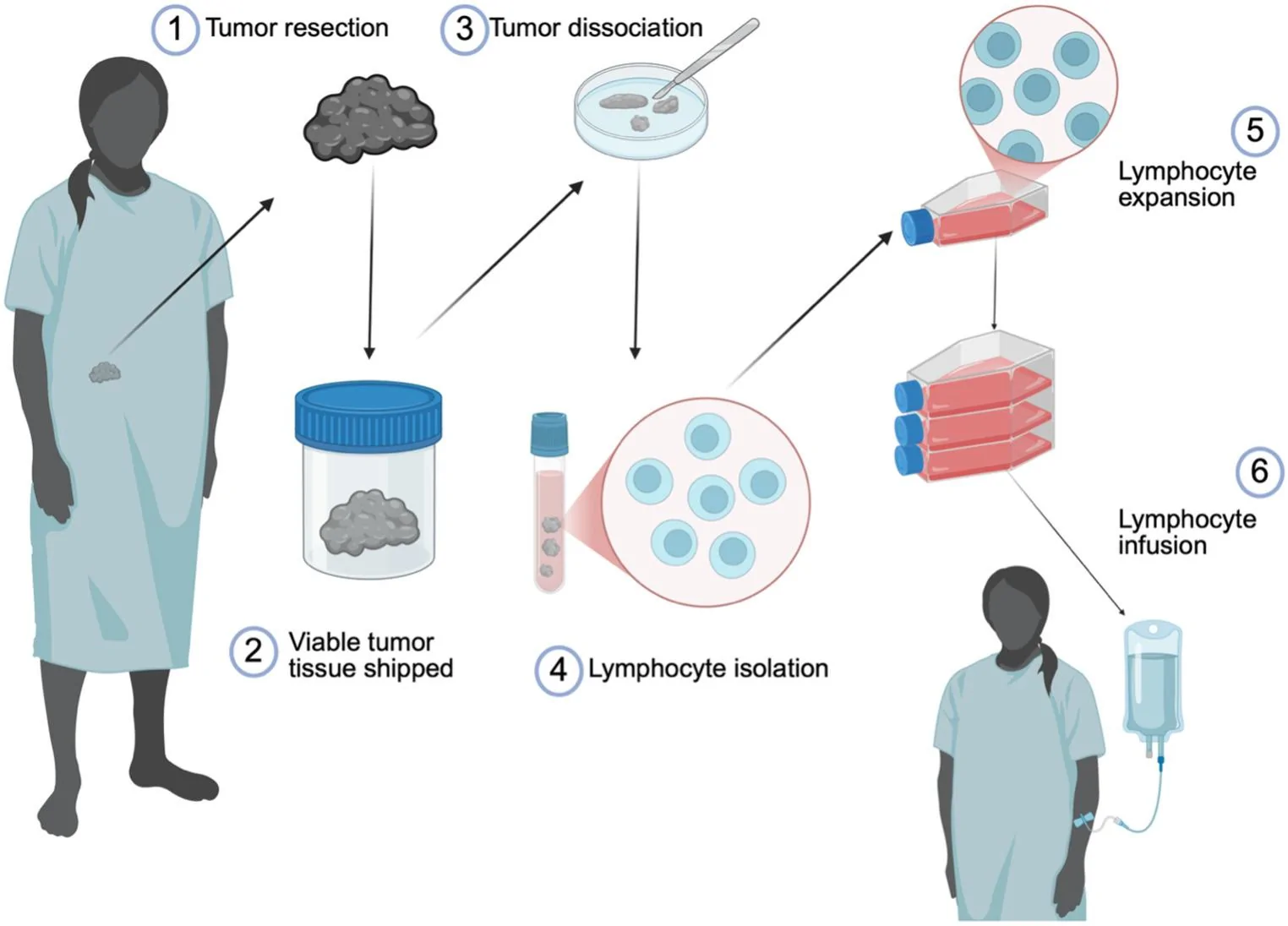
Tissue workflow for TIL isolation. The workflow requires viable tissue made possible by either processing while it is fresh or controlled slow freezing via cryopreservation. Viable lymphocytes extracted autologously from a patient can be expanded ex vivo and reinfused back into the patient as a personalized immunotherapy

Tumor-infiltrating lymphocyte (TIL) therapy extends functional precision medicine from measuring drug response to preserving living immune cells for therapeutic use. Successful TIL manufacturing depends on recovery of viable tumor-reactive lymphocytes (Fig. 3) that retain proliferative capacity, cytokine production, migratory behavior, and cytotoxic function. Preservation strategy therefore affects not only analytical quality, but also the feasibility and potency of the final cellular product.

#### Cellular Recovery and Expansion

Fresh tumor tissue remains the preferred source for TIL isolation because it maximizes initial cell recovery and avoids freeze-thaw injury. However, immediate processing creates substantial logistical constraints related to transport, specialized laboratory access, and centralized manufacturing.

Controlled-rate cryopreservation provides greater flexibility by allowing tumor tissue or isolated lymphocytes to be stored before expansion. Cryopreserved T cells frequently demonstrate post-thaw viability approaching or exceeding 90% and retain the ability to proliferate after thawing [84, 85]. In matched colorectal tumor specimens, cryopreserved tissue supported subsequent T-cell recovery and expansion, although changes in the relative abundance of selected immune-cell populations were observed [86].

Cell recovery may be more sensitive to cryogenic stress than viability alone. Repeated temperature fluctuations during liquid-nitrogen storage produced only modest reductions in PBMC viability but substantially reduced recoverable cell numbers after thawing and overnight culture [87]. Similarly, prolonged cryopreservation has been associated with lower numbers of cells available for sequencing and selective differences in CD4 + and CD8 + T-cell recovery [88, 89]. These findings indicate that evaluation of preserved TIL material should include recovery yield and expansion success in addition to percentage viability.

#### Preservation of Immune Function

The clinical value of preserved TILs depends on maintenance of antitumor function after thawing. In experimental studies, cryopreserved cytotoxic T lymphocytes demonstrated viability comparable to freshly cultured cells and retained activation-marker expression, proliferative capacity, and target-cell killing across conventional and three-dimensional culture assays [84]. Cryopreserved and fresh T cells also exhibited comparable migration through collagen matrices and similar production of interferon-γ and tumor necrosis factor-α following antigen exposure [84].

Nevertheless, functional preservation is not uniform across studies. Cryopreserved T cells isolated from colorectal tumors showed reduced interferon-γ secretion compared with matched fresh-derived cells, despite maintaining the ability to expand after thawing [86]. The timing of cryopreservation may also influence phenotype, with cells frozen after several days of ex vivo expansion showing greater post-thaw phenotypic stability than cells frozen immediately after isolation [85]. FFPE preservation is incompatible with TIL therapy because fixation irreversibly eliminates cellular viability and functional capacity.

#### Clinical Translation

Cryopreservation has become integral to the clinical delivery of TIL therapies because it allows expanded cellular products to undergo release testing, transport, storage, and coordinated infusion. Clinical experience with cryopreserved TIL products demonstrates that viable, tumor-reactive lymphocytes can be manufactured, stored, thawed, and reinfused while retaining therapeutic activity [90].

The possibility of preserving tumor tissue before TIL isolation may further expand access by separating surgical collection from specialized cell-processing facilities. This approach could support centralized manufacturing, multicenter treatment programs, and more flexible processing timelines. However, prospective validation is needed to determine how storage duration, cryoprotectant composition, tissue format, and thawing protocols influence TIL recovery, cellular composition, expansion success, and therapeutic potency.

Collectively, fresh tissue provides the highest initial TIL recovery, while controlled-rate cryopreservation offers the best balance between functional preservation and clinical scalability. Although cryopreservation generally maintains viability and antitumor activity, standardized assessment of cell recovery, phenotype, and potency remains essential.

### T Cell Isolation and CAR-T Therapy and Live Tissue Science

Chimeric antigen receptor T-cell (CAR-T) therapy extends live tissue science into a multistep manufacturing process in which patient-derived T cells are collected, genetically modified, expanded, stored, and reinfused. Successful treatment therefore depends on maintaining cellular viability, proliferative capacity, transgene expression, and antitumor function throughout the manufacturing and distribution workflow.

#### Preservation of Starting Material and Manufacturing Capacity

Fresh leukapheresis material provides high-quality starting cells for CAR-T manufacturing, but immediate processing is often impractical because production may occur at centralized facilities. Cryopreservation allows collection, manufacturing, and treatment scheduling to be separated while supporting transport, quality testing, and repeat processing when needed.

Cryopreserved peripheral blood mononuclear cells and leukapheresis products generally remain suitable for CAR-T manufacturing. Although freezing may reduce the recovery of selected cell populations or slow early expansion, CAR-T cells generated from cryopreserved starting material can retain phenotype, cytokine production, transduction efficiency, and in vitro cytotoxicity comparable to products generated from fresh cells [91, 92]. In a clinical manufacturing cohort, reduced recovery of cryopreserved PBMCs was not associated with lower transduction efficiency, final CAR-T yield, in vitro reactivity, or clinical response [93].

#### Preservation of CAR-T Cell Function

Cryopreservation is also commonly applied to the final engineered cellular product. Controlled-rate freezing with cryoprotective agents such as dimethyl sulfoxide can preserve CAR expression, membrane integrity, cellular phenotype, and target-specific cytotoxicity after thawing. Anti-BCMA CAR-T cells retained transduction rates and major T-cell subsets after cryopreservation, although transient increases in apoptosis and changes in selected functional measurements were observed [94].

Clinical and experimental comparisons indicate that cryopreserved CAR-T products can maintain sufficient expansion and antitumor activity for therapeutic use. CAR-T cells frozen during manufacturing could be thawed, re-expanded to treatment-relevant numbers, and administered successfully, demonstrating that an interrupted manufacturing workflow may remain feasible [93]. Nevertheless, post-thaw evaluation should include viable cell recovery, expansion, phenotype, CAR expression, cytokine production, and cytotoxic potency rather than relying on viability alone.

FFPE preservation is incompatible with CAR-T manufacturing because fixation eliminates cellular viability. FFPE tissue can still support retrospective localization and phenotypic characterization of engineered T cells using specialized in situ hybridization and immunohistochemical methods, but it cannot provide cells for therapeutic production [95]. Experimental rapid-freezing and vitrification approaches may preserve T-cell viability under tightly controlled conditions, but their equipment, warming-rate, and sample-volume requirements currently limit routine manufacturing use [96].

#### Clinical Scalability

Cryopreservation has become central to CAR-T delivery because it enables centralized manufacturing, product release testing, transport, storage, and coordination of lymphodepletion with infusion. It may also provide flexibility when treatment must be delayed or when manufactured cells require additional testing before administration.

Preservation remains only one determinant of product quality. Patient treatment history, lymphocyte fitness at leukapheresis, manufacturing duration, cell composition, storage conditions, thawing procedures, and delays before infusion may also influence the final product. Standardized control of these variables is therefore essential for comparing fresh and cryopreserved workflows.

Collectively, cryopreservation provides the most practical balance between preservation of CAR-T function and the logistical requirements of modern cell-therapy manufacturing. Although modest losses in recovery or transient cellular stress may occur, appropriately controlled cryopreservation generally preserves the manufacturing capacity and antitumor activity required for clinical use.

### Long-Term Storage

Cryopreservation enables long-term preservation of tumor tissue and blood-derived biospecimens at ultra-low temperatures while maintaining molecular and, in some cases, cellular integrity for future downstream analyses. By minimizing enzymatic degradation, nucleic acid fragmentation, and metabolic activity, cryopreservation allows biospecimens to remain suitable for applications including genomic profiling, transcriptomics, proteomics, functional medicine testing, organoid development, and immunotherapy-related research.

The ability to preserve biologically relevant tissue over extended periods has important implications for precision oncology. As therapeutic strategies evolve and resistance mechanisms emerge over time, archived cryopreserved tissue may provide an opportunity for retrospective molecular characterization, identification of newly actionable genomic alterations, therapeutic response modeling, or enrollment into biomarker-driven clinical trials without requiring repeat tissue acquisition. This may be particularly valuable in patients with limited tissue availability or tumors located in anatomically challenging sites where repeat biopsy carries additional procedural risk.

Compared with FFPE preservation, cryopreserved tissue maintains greater molecular fidelity across DNA, RNA, protein, and viable cellular applications. Importantly, preservation of viable cellular populations additionally enables downstream functional analyses that are not feasible using FFPE-derived specimens, including organoid generation, single-cell sequencing, TIL expansion, and functional precision medicine testing. However, successful long-term cryopreservation requires substantial infrastructure, including controlled-rate freezing protocols, liquid nitrogen or ultra-low temperature storage systems, continuous temperature monitoring, and standardized biospecimen handling workflows.

Despite these logistical challenges, the increasing integration of multiomic profiling and live-cell functional assays into precision oncology workflows has expanded interest in prospective biobanking strategies that preserve tissue in states compatible with both current and future molecular applications. As precision medicine continues to evolve, long-term cryopreservation will play an increasingly important role in maintaining clinically and biologically informative biospecimens for translational research and patient-specific therapeutic decision-making.

**Fig. 4:**
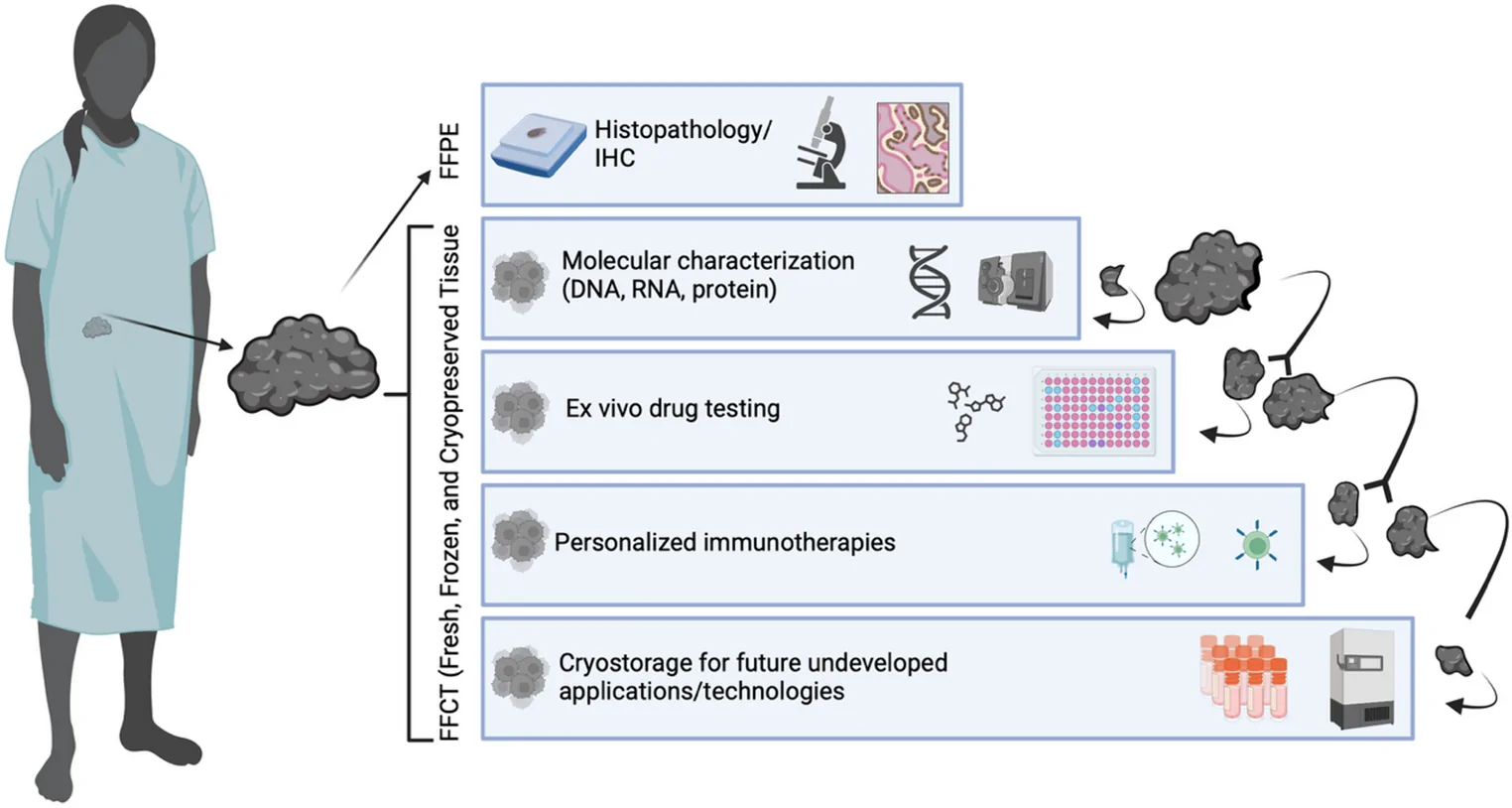
Proposed biospecimen triage strategy. Tumor tissue may be divided among FFPE processing for routine histopathology and fresh, flash-frozen, or cryopreserved formats for molecular profiling, functional precision medicine, personalized immunotherapy development, and future applications. The appropriate allocation depends on tissue availability and the intended downstream use; controlled-rate cryopreservation preserves the broadest range of viable-cell applications

Taken together, these findings support a prospective, tiered approach to biospecimen management rather than reliance on a single preservation method. Tissue required for routine histopathologic diagnosis should be allocated to FFPE, while additional material should be preserved by flash freezing or controlled-rate cryopreservation according to anticipated molecular and functional applications. When adequate tissue is available, cryopreservation retains the broadest range of future options because viable material can subsequently support molecular profiling, functional precision medicine, organoid development, and cell-based therapeutic research. A proposed framework for this specimen-allocation strategy is shown in Fig. 4.

## Conclusions

The evidence reviewed here demonstrates that tissue preservation is not merely a pre-analytical consideration but a fundamental determinant of molecular and functional data quality. As precision oncology increasingly incorporates genomic, transcriptomic, proteomic, epigenetic, and live-cell analyses into clinical decision-making, biospecimen management must evolve beyond workflows developed primarily for histopathologic evaluation. A practical approach is to prospectively triage tumor specimens according to their intended downstream applications by allocating sufficient tissue for routine FFPE diagnosis while preserving additional material by flash freezing or controlled-rate cryopreservation for molecular and functional analyses. This strategy has already been successfully implemented in several institutions, including more than half of cancer centers in the Netherlands where FF tissue is prioritized for whole-genome sequencing [40].

Although substantial advances have improved the analytical performance of FFPE-derived specimens, many of these improvements rely on increasingly sophisticated extraction protocols, library preparation methods, and bioinformatic correction strategies designed to compensate for preservation-induced molecular damage, a practice referred to as rescue science. The collective evidence suggests that comparable or greater improvements in molecular fidelity may be achieved by optimizing biospecimen collection and preservation before analytical testing begins. Importantly, this does not diminish the indispensable role of FFPE in routine pathology; rather, it emphasizes that preservation strategy should be matched to the intended clinical or research application and should be considered part of assay design rather than as an independent pre-analytical variable.

Ultimately, the value of molecular characterization lies not in generating technically reproducible data alone, but in producing clinically meaningful information that improves patient care. Regardless of preservation method, molecular assays must demonstrate clinical validity before they can meaningfully guide therapeutic decision-making [97]. As this review demonstrates, preservation-associated alterations become progressively more consequential as molecular analyses advance from DNA sequencing to RNA profiling, proteomics, and ultimately to functional live-cell applications. Accordingly, preservation methods that maintain higher molecular fidelity are likely to provide greater clinical utility for applications requiring quantitative molecular measurements or preservation of viable cellular function.

The emergence of organoid models, functional precision medicine testing, single-cell sequencing, TIL therapies, and CAR-T manufacturing further expands the role of biospecimen preservation beyond molecular characterization alone. Controlled-rate cryopreservation has consistently demonstrated the closest approximation to fresh tissue across many of these downstream applications while providing the flexibility required for longitudinal biobanking and multicenter precision oncology programs [55]. Rather than adopting a single preservation strategy for every specimen, future precision oncology workflows will likely benefit from tiered biospecimen preservation approaches that balance diagnostic pathology, molecular fidelity, preservation of viable cellular function, logistical feasibility, and long-term clinical utility. In this framework, FFPE remains advantageous for routine histopathology and morphology-based assessment, fresh tissue for immediate functional interrogation, flash-frozen tissue for rapid preservation of molecular analytes, and controlled-rate cryopreservation when retention of viable tissue and future functional applications are priorities. Such an approach recognizes that the optimal preservation method depends not only on the tissue itself, but also on the biological questions and therapeutic decisions the tissue is intended to support.

## Key References

- Basyuni S, Heskin L, Degasperi A, Black D, Koh GCC, Chmelova L, et al. Large-scale analysis of whole genome sequencing data from formalin-fixed paraffin-embedded cancer specimens demonstrates preservation of clinical utility. Nat Commun. 2024;15:7731.https://doi.org/10.1038/s41467-024-51577-2.
  - This large-scale study demonstrates that clinically useful whole-genome sequencing can be achieved from FFPE cancer specimens while also defining the characteristic sequencing artifacts and quality limitations introduced by formalin fixation.
- Dixon K, Lee JH, Miller R, Booker D, Anderson D, Okojie J, et al. Cryopreserved Tissue Biospecimens Offer Superior Quality for Whole-Genome Sequencing of Various Cancers Compared to Paired Formalin-Fixed Paraffin-Embedded Tissues. Int J Mol Sci. 2025;26(22):11038. https://doi.org/10.3390/ijms262211038.
  - This matched pan-cancer study directly demonstrates improved DNA integrity, library quality, and whole-genome sequencing performance in cryopreserved tissue compared with paired FFPE specimens.
- Chen P, Zhou JB, Chu XP, Feng YY, Zeng QB, Lei JH, et al. Establishing a cryopreserved biobank of living tumor tissues for drug sensitivity testing. Bioact Mater. 2025;46:582-596.https://doi.org/10.1016/j.bioactmat.2024.09.008.
  - This work demonstrates the feasibility of banking viable tumor tissue for later functional drug-sensitivity testing and highlights the expanding role of cryopreservation beyond molecular profiling into functional precision medicine.
- Jacobsen SB, Tfelt-Hansen J, Smerup MH, Andersen JD, Morling N. Comparison of whole transcriptome sequencing of fresh, frozen, and formalin-fixed, paraffin-embedded cardiac tissue. PLoS One. 2023;18(3):e0283159.https://doi.org/10.1371/journal.pone.0283159.
  - This direct comparison of fresh, frozen, and FFPE tissue demonstrates how preservation method influences RNA integrity and whole-transcriptome sequencing performance, providing important evidence that tissue storage can substantially affect transcriptomic fidelity.
- Restivo G, Tastanova A, Balázs Z, Panebianco F, Diepenbruck M, Ercan C, et al. Live slow-frozen human tumor tissues viable for 2D, 3D, ex vivo cultures and single-cell RNAseq. Commun Biol. 2022;5:1144.https://doi.org/10.1038/s42003-022-04025-0.
  - This study demonstrates that slow-frozen human tumor tissue can retain sufficient viability and biological fidelity for 2D and 3D culture, ex vivo analysis, and single-cell RNA sequencing, making it a key example of preservation enabling both molecular and functional applications.
- Boilève A, Cartry J, Goudarzi N, Bedja S, Mathieu JRR, Bani MA, et al. Organoids for Functional Precision Medicine in Advanced Pancreatic Cancer. Gastroenterology. 2024;167:961-976.e13.https://doi.org/10.1053/j.gastro.2024.05.032.
  - This prospective study demonstrates the clinical feasibility of organoid-based drug screening and illustrates how preservation of viable tumor tissue can support functional precision medicine.

## Data Availability

No datasets were generated or analysed during the current study.

## Abbreviations

CAR-T: chimeric antigen receptor T cell
FF: flash frozen
FFCT: fresh, flash-frozen, cryopreserved tissue
FFPE: formalin-fixed, paraffin-embedded
NGS: next generation sequencing
TIL: tumor-infiltrating lymphocyte
VAF: variant allele frequency
WGS: whole genome sequencing

## Abbreviations

CAR-T: chimeric antigen receptor T cell
FF: flash frozen
FFCT: fresh, flash-frozen, cryopreserved tissue
FFPE: formalin-fixed, paraffin-embedded
NGS: next generation sequencing
TIL: tumor-infiltrating lymphocyte
VAF: variant allele frequency
WGS: whole genome sequencing
